# Spatial immune niche remodeling of the neutrophil-macrophage axis inchronic liver disease

**DOI:** 10.3389/fcell.2026.1902627

**Published:** 2026-08-14

**Authors:** Jian Ding, Xiang Xiong, Changming Zhang, Hongcai Fang, Shu Li

**Affiliations:** 1 The Affiliated Hospital of Jiujiang University, Jiujiang, China; 2 Eastern Theater Command General Hospital of the People’s Liberation Army, Nanjing, China

**Keywords:** cell crosstalk, chronic liver disease, immune microenvironment, liver fibrosis, macrophages, neutrophils

## Abstract

Chronic liver disease (CLD) arises from diverse etiologies, featuring persistent inflammation, progressive fibrosis and eventual hepatocellular carcinoma (HCC). Dynamic remodeling of the hepatic immune microenvironment dominates CLD progression. As core innate immune cells, neutrophils and macrophages form a critical crosstalk hub linking inflammatory response and tissue remodeling. The 2 cell populations interact bidirectionally *via* direct cell contact and soluble mediators including cytokines, chemokines, extracellular vesicles and neutrophil extracellular traps (NETs), building a complicated regulatory network. This network mediates hepatic stellate cell activation, fibrotic progression and tumor immune escape as sterile inflammation exacerbates. Mechanistically, neutrophil-derived NETs and reactive oxygen species (ROS) trigger macrophage nuclear factor-κB (NF-κB) and NOD-like receptor family pyrin domain-containing 3 (NLRP3) inflammasome signaling. Macrophages reciprocally secrete chemokines to recruit neutrophils and modulate their functional phenotypes. Notably, this crosstalk exhibits stage-dependent effects: it amplifies inflammation at early disease phases yet drives immunosuppression and tissue remodeling at late stages. This review systematically addresses neutrophil and macrophage phenotypic heterogeneity and their interactive mechanisms in CLD, highlighting stage-specific spatiotemporal functional reprogramming and summarizing targeted therapeutic strategies. Elucidating this core immune axis lays novel theoretical foundations and therapeutic targets for precision immune intervention against CLD.

## 1 Introduction

Chronic liver disease, including viral hepatitis, metabolic dysfunction-associated steatohepatitis (MASH), alcohol-associated liver disease (ALD) and autoimmune liver disease, is an important public health problem that leads to increased morbidity and mortality worldwide (De Siervi et al., 2023). Although the etiology is diverse, different types of CLD show relatively consistent pathological changes in pathological progression, that ischronic inflammatory response caused by persistent liver cell injury, which in turn drives the activation of hepatic stellate cells and the deposition of extracellular matrix (ECM), and finally develops into cirrhosis and even hepatocellular carcinoma (Yan et al., 2025).

Most of the previous studies focused on the mechanisms of liver cell injury and hepatic stellate cell (HSC) activation. However, in recent years, more and more research evidence has shown that the liver immune microenvironment is the key factor in the progression of the disease (Deng et al., 2025). The liver continuously receives antigens and various metabolites from the intestine through the portal vein to maintain a dynamic balance between immune tolerance and immune activation. Once the balance is broken, the liver immune microenvironment will be disordered, which is also one of the core causes of the continuous progress and deterioration of chronic liver disease (Tao et al., 2025).

In this complex microenvironment, neutrophils and macrophages constitute the core cell population of innate immune defense (Mihlan et al., 2024). In the past, we generally believed that neutrophils have a short life span and are mainly involved in acute inflammatory responses; the role of macrophages is mostly responsiblefor engulfing and cleaning up cell debris, as well as antigen presentation. However, with the maturityofnew technologies such as single cell sequencing and spatial transcriptome, it has been found that these two kinds of cells actually have obvious phenotypic differences, and their functions also have strong plasticity, which is not only as single as we previously knew (Garrido-Trigo et al., 2023). In the state of chronic inflammation, thesetwo kinds of cells are not only involved in immune defense so simple, but also deeply involved in theprocess of tissue damage, fibrosis, and tumor immune regulation, which plays a very important role (Xue et al., 2022).

More importantly, the latest research has found that there is a wide range and particularly complexinteraction between neutrophils and macrophages. They can communicate in several ways, such as directcell contact, various cytokine linkage, metabolic state change, and structural molecules such as NETs, which together form the core of the regulatory network that is very critical in regulating the liver immune microenvironment (Wang Y. et al., 2024). This set of regulatory networks is not static in different stages of disease development, but will be dynamically adjusted with changes in the disease. It has a profound impact on which direction the disease will eventually develop and how the outcome will be. It is a key factor that cannot be ignored.

Therefore, this article systematically reviews the core biological characteristics of neutrophils and macrophages in chronic liver disease, focuses on the spatiotemporal interaction mechanism of the two types of cells and their functional reprogramming that dynamically changes with the course of disease, andexplores potential intervention strategies around this key immune axis, in order to provide a new theoretical basis for precise immunotherapy of CLD.

At present, many classic reviews have summarized the overall characteristics of liver innate immunity, and some studies have summarized various phenotypes of myeloid cells. For example, the study published in ‘Clinical Science’ in 2021 systematically sorted out the related systems of liver immune microenvironment. However, in general, the research framework of the existing review is relatively simple. Different from the previous research methods that only simply summarize cell markers, this paper constructs a new analysis idea and research system. Based on the complete disease progression sequence of chronic liver disease, the characteristics and functional remodeling of neutrophil-macrophage axis in different stages of chronic liver disease were systematically explained, covering the complete pathological process of acute onset of disease, chronic fibrosis matrix remodeling and late tumor immune escape. At the same time, this study further combines the sequential dynamic changes of the immune axis with the pathogenesis of the disease, the limitations of current experimental techniques and the difficulties of clinical treatment, and establishes a research framework with both theoretical value and clinical transformation potential, which can provide practical theoretical reference and research and development ideas for precise targeted intervention strategies of myeloid cells at different stages of disease.

## 2 Neutrophil heterogeneity

### 2.1 Recruitment and spatial distribution of neutrophils

Studies have confirmed that in the early stage of chronic liver disease, a large number of neutrophils migrate to liver tissue, which is the key to induce local inflammation, and the whole recruitment process is regulated by multiple signaling pathways. When liver cells are damaged and the body‘s metabolic homeostasis is unbalanced, cells will release a large number of damage-related molecular patterns (DAMPs). These molecules can activate the Toll-like receptor (TLRs) signaling pathway, further act on the liver Kupffer cells and hepatic sinusoidal endothelial cells, and finally amplify the inflammatory signal step by step, opening the inflammatory cascade (Porfírio do Nascimento et al., 2025). Under this premise, the damaged liver cells and immune cells in the liver itself, will secrete CXCL1 (C-X-C motif chemokine ligand 1), CXCL2 (C-X-C motif chemokine ligand 2), CXCL8 (C-X-C motif chemokine ligand 8) also known as IL-8 (Interleukin-8) such chemokines. These factors can bind to the CXCR2 receptor on the surface of neutrophils to form a classic CXCR2 chemotaxis regulation pathway, which in turn promotes the activation of neutrophils inbone marrow to be released into the blood, and then migrate to the site of liver injury (Liu et al., 2024).

In fact, this recruitment process of neutrophils is not only completed in local liver tissues, but alsoinvolves a set of systemic regulatory mechanisms of bone marrow-peripheral blood-lesion organs. Inflammation signals the granulocyte colony-stimulating factor (G-CSF), and other hematopoietic-related factors to enhance the ability of bone marrow to generate granulocytes, thereby increasing the number of neutrophils in the body (Bun et al., 2024). In addition, sympathetic nerves and circadian rhythms have also been confirmedto be involved in regulating the efficiency of neutrophil mobilization. This also shows that the recruitment process of neutrophils is not controlled by a single factor, but a very complex whole body regulation mechanism (Li H. et al., 2025).

Neutrophils in peripheral tissues usually follow the classic step-by-step recruitment model of ‘rolling, adhesion, migration’, but the structure of liver microcirculation is special. The shear force generated by the blood flow in the hepatic sinus region is small, and neutrophils do not need to complete the rolling steps regulated by selectin, and can quickly stay in the liver tissue and migrate directly with the help of integrins and various adhesion molecules (Zhang N. et al., 2024). Specifically, CD44 and Mac-1 (CD11b/CD18) expressed on the surface of neutrophils can bind to hyaluronic acid (HA) and intercellular adhesion molecule-1 (ICAM-1) in tissues, respectively. Based on this interaction, neutrophils can be captured by the sinusoidal endothelium, firmly adhere, and then migrate through the endothelial cells (Iqbal et al., 2022; McDonald et al., 2010). In addition, the inflammatory environment also activates hepatic sinusoidal endothelial cells, promotes the expression of adhesion molecules such as ICAM-1 and Vascular Cell Adhesion Molecule-1 (VCAM-1)on the cell surface, and changes the glycocalyx structure of the endothelial surface, which will further enhance the adhesion ability of neutrophils (Akbar et al., 2023).

Many research groups have carried out related research through spatial transcriptome and single cell sequencing technology. The results showed that the distribution of neutrophils in the liver had obviousregional preference, mostly concentrated in the tissue necrosis area, lipid deposition site and the periphery of inflammatory lesions in the portal area (Kim and Lachmann, 2026).

The characteristics of neutrophil aggregation in the liver are mainly regulated by local chemokine concentration gradient, tissue oxygen partial pressure and metabolic state.Taking the hypoxic microenvironment as an example, this environment can activate the Hypoxia-Inducible Factor 1 alpha (HIF-1α) pathway. On the one hand, it enhances the chemotaxis of neutrophils, on the other hand, it prolongs cell survival time, and ultimately causes neutrophils to accumulate in a specific microenvironment (Nakamura et al., 2024).

### 2.2 Effector function: from anti-infective defense to aseptic inflammation drive

In the microenvironment of chronic liver disease, the function of neutrophils has changed significantly. Neutrophils are no longer limited to traditional anti-infective defense, but participate in the regulation of aseptic inflammatory response as important effector cells (Xue et al., 2022; Huang et al., 2023). Neutrophils mainly rely on degranulation, production of reactive oxygen species, and release of neutrophil extracellular traps. Although such reactions can protect the body, they can also cause liver tissue damage (Willson et al., 2022).

Neutrophils will release myeloperoxidase (MPO), neutrophil elastase (NE), cathepsin G and other granular proteins, and can also produce a large amount of reactive oxygen species by Nicotinamide Adenine Dinucleotide Phosphate (NADPH)oxidase system, which will eventually cause oxidative stress damage to liver cells and cause lipid peroxidation (Tang et al., 2024). At the same time, reactive oxygen species can also act as a signal molecule to regulate inflammation-related pathways, which can promote the assembly and activation of inflammasomes, so that the local inflammatory response continues to increase (Bang et al., 2021).

In recent years, the generation mechanism of NETs has attracted wide attention. The structure of NETs is composed of depolymerized chromatin as a scaffold, combined with a variety of antibacterial proteins; Peptidyl Arginine Deiminase 4 (PAD4)catalyzes the citrullination of histones and promotes chromatin depolymerization, which is an essential part of NETs formation (Shahzad et al., 2025). In the process of chronic liver disease, the body often generates a large number of NETs, and such structures are difficult to be cleared in time, which ultimately causes obvious pathological damage to liver tissue. On the one hand, NETs can be recognized by TLR4, TLR9 and other receptors on the surface of macrophages as a damage-related molecular pattern, activating Nuclear Factor kappa-light-chain-enhancer of activated B cells (NF-κB) and NLRP3 inflammasome pathways; on the other hand, the protease and DNA structure in NETs can directly stimulate hepatic stellate cells, induce their phenotypic transformation to myofibroblasts and promote collagen deposition (Zeng et al., 2025).

In addition, neutrophils can also regulate the recruitment and activation of other immune cells by releasing cytokines and chemokines (such as IL-17, CXCL10, *etc.*), thus playing an “amplifier” role inthe immune network. It is worth noting that in the continuous inflammatory environment, the clearance function of neutrophils decreases, resulting in the persistence of inflammatory signals and the formation of chronic inflammatory state (Jia et al., 2023).

### 2.3 Phenotypic heterogeneity and functional plasticity with the development of single cell

Sequencing and multi-omics technology, neutrophils have been proved to have significant phenotypic heterogeneity and functional plasticity (Lee et al., 2025). In different stages of chronic liver disease and different microenvironments, neutrophils can differentiate into a variety of functional specific subsets, and their phenotype and function are regulated by local inflammatory signals, metabolic status and intercellular interactions (Wang and Liu, 2021).

In the context of pro-inflammatory and pro-fibrotic, some neutrophils show the characteristics of high expression of PAD4, which plays a central role in the formation of NETs (Liu H. et al., 2025). PAD4-dependent NETs release is considered to be an important mechanism linking inflammation and hepatic stellate cell activation, and its excessive activation can promote inflammation amplification and fibrosis progression through TLR and NLRP3 inflammasome pathways (Yanginlar et al., 2024). In addition, some studies have also found that specific neutrophil subsets can express higher levels of C-X-C Chemokine Receptor 4 (CXCR4), showing prolonged lifespan and enhanced tissue retention, thereby exacerbating chronic inflammation (Ngamsri et al., 2021).

On the contrary, in the stage of inflammation regression and tissue repair, neutrophils can be transformed into phenotypes with anti-inflammatory and repair functions. For example, some neutrophils can participate in the degradation of extracellular matrix by secreting matrix metalloproteinases, and promote the reversal of fibrosis (Sabir et al., 2023); At the same time, the released extracellular vesicles (especially rich in miR-223) can inhibit the activation of macrophage inflammasomes, thereby promoting inflammation regression (Gu et al., 2022).

Neutrophils will participate in immune regulation after entering the tumor microenvironment, and can further differentiate into polymorphonuclear myeloid-derived suppressor cells (PMN-MDSCs) (Wang C. et al., 2023). These cells can express Arginase 1 (Arg-1), produce reactive oxygen species, active nitrogen, and consume local nutrition of the lesion, thereby inhibiting the activity of CD8 positive T cells and weakening the body‘s own anti-tumor immunity. In addition, PMN-MDSCs can also secrete various pro-angiogenic factors and reshape the tissue matrix structure to further accelerate tumor development (Liao et al., 2025). At present, our understanding of the mechanism of neutrophil function remodeling is mostly derived from animal model studies. However, recent human clinical datasets and single-cell maps have revealed highly specialized, microenvironment-specific neutrophil activation states that drive active tissue remodeling. A prominent paradigm is represented by ductular reaction-associated neutrophils (DRANs), a human-validated active neutrophil subset that preferentially accumulates within periportal niches during chronic liver diseases. Rather than acting as transient bystander inflammatory cells, DRANs physically and biochemically align with the portal tract. Emerging evidence demonstrates that DRANs recruitment and microenvironmental synchronization directly promote progressive ductular reaction and biliary epithelial proliferation (Ariño et al., 2023). This result highlights that neutrophils play an active and irreplaceable role in mediating the progressive remodeling of biliary tract and severe tissue fibrosis in patients. At the same time, it is confirmedthat the phenotype of neutrophils in human liver diseases is not static, but will continue to undergo dynamic and highly plastic functional remodeling. In addition to the various neutrophil subtypes differentiated by cell density, more and more studies have confirmed that low-density neutrophils (LDNs) and neutrophil epigenetically regulated training immunity can continuously maintain the level of systemic inflammation and promote tumorigenesis, which has high clinical research value (Zhang et al., 2025). When centrifuged and stratified, conventional high-density neutrophils can be separated from monocytes, while LDNs are mixed with monocytes in the same component. These cells have unique transcription and secretion characteristics, either have a stronger immunosuppressive effect, or cause more significant inflammatory damage. When centrifuged and stratified, conventional high-density neutrophils can be separated from monocytes, while LDNs re mixed with monocytes in the same component. These cells have unique transcription and secretion characteristics, either havea stronger immunosuppressive effect, or cause more significant inflammatory damage. The existing chromatin open region sequencing studies have confirmed that cells can maintain such special functions for a long time, which is rooted in the deep changes in the chromatin structure and epigenetic regulatory network of neutrophils. When the body has systemic inflammatory stimulation, or local tissue metabolic disorders, neutrophil progenitor cells in the bone marrow will form stable histone modifications (such asH3K4me1, H3K27ac enrichment), while changing the regulatory mode of transcription factors (Kalafati et al., 2023). After this kind of epigenetic reprogramming process, mature neutrophils will retain pathogenic memory for a long time, and their plasticity and transcriptional response threshold will continue to change. These cells have been pre-differentiated into a specific subtype of high inflammatory response and pro-tumor even before migrating to the liver microenvironment (Jiao et al., 2026).

Based on the above content, it is not difficult to see that neutrophils in the liver do not have a unified cell phenotype, but a set of dynamic cell lineages that can adjust their functions according to the pathological environment. In order to clearly distinguish the different states of various types of neutrophils, we systematically summarized the markers of major neutrophil subtypes in chronic liver diseases, induced stimulation signals, and corresponding pathological damage to Table 1.

**TABLE 1:** Phenotypic and functional heterogeneity of neutrophils in CLD.

Subtype	Key markers	Inducing signals	Major functions	Pathological role
Pro-inflammatory neutrophils	CD177^high^, LOX-1^+^, CD62L^low^, CXCR2^+^	DAMPs, IL-8, CXCL1/2	ROS production, degranulation, high cytotoxicity	Amplify sterile inflammation, drive initial hepatocyte injury
NETs-forming neutrophils	PAD4^+^	ROS, inflammation	NETs release	Promote fibrosis
CXCR4^+^ aged neutrophils	CXCR4^+^	Chronic inflammation	Prolonged survival	Sustain inflammation
Pro-resolving neutrophils	MMPs^+^	Resolution signals	ECM degradation	Repair
PMN-MDSCs	Arg-1^+^	Tumor microenvironment	T cell suppression	Immune evasion

## 3 Macrophage heterogeneity

### 3.1 The origin and lineage remodeling of hepatic macrophages

The liver is one of the organs with the highest degree of macrophage enrichment in the human body. Macrophages are divided into two groups. One is Kupffer cells (KCs) colonized in the liver during embryonic development, and the other is differentiated from peripheral circulating monocytes (Guilliams and Scott, 2022). In recent years, relevant studies have found that the commonly used M1 and M2 polarization division in the past has been unable to fully describe the diverse functional performance of macrophages in chronic liver diseases. Nowadays, it is generally believed that macrophages will continue to undergo lineage remodeling with cell origin, local liver microenvironment and various signal stimuli, and this dynamic change is the core research focus (Roohani and Tacke, 2021).

Under normal physiological conditions, Kupffer cells formed in the embryonic stage are produced from the yolk sac or fetal liver hematopoietic tissue. After the fetus is born, these cells can proliferate and renew themselves in the hepatic sinus and remain stable for a long time (Sheu and Hoffmann, 2022). Whether these cells can survive normally and exert physiological functions depends entirely on various signals released by the local microenvironment of the lesion. The secretion of Notch ligands by sinusoidal endothelial cells, the release of various metabolic factors by hepatocytes, and the addition of transforming growth factor β are all key signaling molecules that regulate this process (Yang S. et al., 2025). The co-regulation of multiple signals allows Kupffer cells to maintain an immune tolerance state, which can not only remove invasive pathogens and apoptotic cells, but also avoid excessive activation of the inflammatory response. KCs usually highly express marker molecules such as C-type lectin domain family 4 member F (CLEC4F), T cell immunoglobulin and mucin domain-containing protein 4 (TIM4^+^) and V-set and immunoglobulin domain containing 4 (VSIG4), and show certain spatial distribution characteristics in hepatic lobules (Pessenda et al., 2025; Liu J. et al., 2025).

During the progression of chronic liver disease, continuous inflammatory stimulation, lipid toxicity and oxidative stress can lead to functional exhaustion, phenotypic changes and even apoptosis of primitive KCs, thus destroying their homeostasis. At the same time, Ly6C^high inflammatory monocytes in peripheral blood are recruited into the liver and differentiate into monocyte-derived macrophages (MoMFs) driven by the CCL2-CCR2 axis. In the early stage, most of these cells exhibit pro-inflammatory phenotype and secrete inflammatory factors such as TNF-α and IL-1β, thereby amplifying the local inflammatory response (Sun et al., 2022).

It is worth noting that MoMFs are not terminally differentiated, but exhibit a high degree of plasticity under the regulation of local microenvironment signals,such as Notch, Liver X Receptor (LXR)and Transforming Growth Factor-beta (TGF-β) signals (Zhang S. et al., 2023). Some MoMFs can gradually obtain phenotypic characteristics similar to KCs, forming the so-called monocyte-derived KCs. However, these cells are still different from embryonic-derived KCs in terms of transcriptome and function, and their immune tolerance is usually insufficient, which plays an important role in the maintenance of chronic inflammation (Lu et al., 2024).

### 3.2 Mechanism of metabolic reprogramming and inflammation amplification

In the microenvironment of chronic liver disease, macrophages not only change their phenotypic characteristics, but also undergo great remodeling of their immune metabolism. Macrophages differentiated from monocytes, after being stimulated by inflammation, the way of energy metabolism will change from the original mainly relying on mitochondrial oxidative phosphorylation to aerobic glycolysis. This type of metabolic transformation is very similar to the Warburg effect, which can not only quickly supply energy to support cells to cope with inflammatory stimuli, but also synthesize various raw materials forcell proliferation (Gao B. et al., 2025).

After the metabolism of phagocytic cells is remodeled, the glycolysis reaction is enhanced, the tricarboxylic acid cycle is blocked, and a large number of metabolic intermediates are retained in the cells. Taking succinic acid as an example, the accumulated succinic acid can stabilize HIF-1α and continuously promote the expression of pro-inflammatory factors such as IL-1β. At the same time, the intracellular citric acid flows out. which can also provide raw materials for lipid synthesis and the production of various inflammatory mediators. There is a close linkage between metabolic pathways and inflammatory signals. Relying on this set of regulatory mechanisms, macrophages can maintain a pro-inflammatory activation state for a long time (Wu et al., 2024).

Lipid metabolism disorder is an important incentive to promote the continuous progress of chronic liver disease. After macrophages ingest free fatty acids, cholesterol crystals and oxidized lipids, they will simultaneously trigger dual stress of lysosomes and endoplasmic reticulum, thereby activating NLRP3 inflammasome and exacerbating local inflammation (Pan et al., 2024). At the same time, the abnormal function of cholesterol transporters such as ATP-binding cassette transporter A1 (ABCA1) and ATP-binding cassette transporter G1 (ABCG1) will also aggravate lipid accumulation and further amplify the inflammatory response (Laguna-Maldonado et al., 2025).

The hypoxic microenvironment is also an important factor in regulating macrophage metabolism. In chronic liver disease, the destruction of hepatic sinus structure and blood flow disorder can lead to local hypoxia. The activation of HIF signal not only promotes glycolysis, but also regulates macrophage chemotaxis and inflammatory gene expression (Bai et al., 2025). In addition, mitochondrial dysfunction and ROS accumulation are also involved in the regulation of inflammatory response, forming a positive feedback loop of metabolism-oxidative stress-inflammation (Xu et al., 2025).

### 3.3 Disease-specific macrophage subsets

With the development of single cell sequencing and spatial transcriptome technology, the academic community has found macrophage subtypes with different functions in different stages of chronic liver disease. These cells are involved in the regulation of the whole process of the disease, and their distribution in the liver tissue is fixed, and each bears different pathological functions (Li Y. et al., 2025).

In metabolically related steatohepatitis, lipid-associated macrophages (LAMs) are the most studied representative subtype. These cells highly express Triggering Receptor Expressed on Myeloid Cells 2 (TREM2), CD9 and other markers, concentrated in the area of lipid accumulation and liver cell damage, arranged in a similar coronal structure. In the early stage of the disease, LAMs can engulf excess lipid and cell debris, which has a protective effect on the liver (Xu et al., 2024); If lipids continue to accumulate, cholesterol will form crystals, and lipotoxicity will continue to stimulate, which will destroy lysosomes and activate NLRP3 inflammasomes, allowing LAMs to play a pro-inflammatory and pro-fibrotic role, thus reflecting the two-way and phased functional characteristics of the cells (Kui et al., 2024).

After the liver develops to the stage of fibrosis, scar-associated macrophages (SAMs) will play a key regulatory role. These cells are abundantly distributed in the fibrous septum, highly expressing CD9 and TREM2, and are closely adjacent to activated hepatic stellate cells (Fabre et al., 2023). On the one hand, SAMs secrete TGF-β and platelet-derived growth factor (PDGF), on the other hand, they directly induce the activation of hepatic stellate cells and promote the deposition of collagen by Notch and Wnt signaling pathways, which are the core cells to promote the sustainable development of liver fibrosis (Huang et al., 2024).

In addition, macrophage subsets with repair function can also be observed in the inflammatory regression stage. These cells usually upregulate matrix metalloproteinases (MMPs) and anti-inflammatory factors (such as IL-10), participate in extracellular matrix degradation and tissue remodeling, and are of great significance in the process of fibrosis reversal (Duan et al., 2022).

In the stage of tumorigenesis, macrophages further differentiate into tumor-associated macrophages (TAMs) (Zhu et al., 2025). Most of these cells have an immunosuppressive phenotype, which can secrete IL-10 and express PD-L1 molecules, while consuming the local nutrition of the lesion and reshaping the metabolic microenvironment, thereby weakening the anti-tumor effect of T cells (Pu and Ji, 2022). In addition, tumor-associated macrophages can secrete pro-angiogenic factors such as Vascular Endothelial Growth Factor (VEGF) and matrix remodeling proteases, induce tumor angiogenesis, and promote tumor invasion and proliferation (Zhao et al., 2022). It is worth emphasizing that a large number of human single cell sequencing and multi-omics panoramic mapping studies have fully proved the real existence of the above-mentioned macrophage subtypes in the human body, making the achievements in this field have higher clinical transformation value. The classic human liver single cell dataset built by the MacParland et al. (2018), Ramach et al. (2019), and Aizarani et al. (2019) have studies have clarified the corresponding immune microenvironment localization of various myeloid cells with the help of clinical patient samples. Among them, Ramachandran et al., used the clinically resected human cirrhotic liver tissue to completely characterize the transcription profile of SAMs in the fibrous septum, confirming that these cells highly express TREM2 and CD9 in the patient‘s tissue. In addition, human high-throughput single-cell sequencing for metabolic liver disease has also made many discoveries—most notably exemplified by the human MASH and Non-Alcoholic Fatty Liver Disease (NAFLD) landscapes dissected by Wang et al. (2019)—study was relied on human samples to complete the verification. It was observed that a large number of lipid-associated macrophages LAMs were enriched in the area of liver coronary lesions, which directly confirmed that these cells were involved in the stress response caused by lipid accumulation and mediated local metabolic inflammation. Major macrophage subsets and their functional characteristics are summarized in Table 2.

**TABLE 2:** Macrophage subsets in CLD.

Subtype	Origin	Key markers	Localization	Function	Stage
Kupffer cells	Embryonic	CLEC4F^+^	Sinusoids	Tolerance	Early
MoMFs	Monocytes	Ly6C^high	Injury sites	Inflammation	Early
LAMs	MoMFs	TREM2^+^	Lipid regions	Lipid handling	MASH
SAMs	MoMFs	CD9^+^	Fibrotic areas	Fibrosis	Fibrosis
TAMs	Mixed	PD-L1^+^	Tumor	Immunosuppression	HCC

## 4 Spatial niche-dependent crosstalk

The basic functional unit of the liver is the hepatic lobule. Such units are repeatedly distributed in the liver, and the internal metabolic system and vascular arrangement are very regular. Blood enters the hepatic lobule through the portal area composed of portal vein, hepatic artery and bile duct, passes through the hepatic sinus, and finally flows to the central vein (Kim and Lachmann, 2026). The unidirectional flow of blood will form a gradient along the axis from the portal to the central vein, and the concentration of oxygen, nutrition, intestinal microbial metabolites and various metabolic hormones in the liver will gradually change. Affected by this blood flow gradient, the liver parenchyma can be divided into three functional zones: Zone 1 is the periphery of the portal, with sufficient oxygen and nutrition supply; Zone 2 is the middle area of hepatic lobule; Zone 3 is close to the central vein, with insufficient oxygen supply and moremetabolic waste accumulation (Aizarani et al., 2019). New technologies such as high-resolution spatial transcriptome, spatialmulti-omics, and multiple ion beam imaging have shown that this partition structure of the liver will affect neutrophils and macrophages in chronic liver diseases from the root: the cell phenotypes of the two are different, the tissue distribution location is different, and the signal exchange between cells is also different.

### 4.1 Metabolic gradients and spatial seeding of immune niches

The oxygen partial pressure in Zone 1 is about 60–65 mmHg, and that in Zone 3 is only 30–35 mmHg. The oxygen levels in the two areas are very different, forming two completely different physiological microenvironments, which have differential regulation on immune cells. Area 1 has sufficient oxygen, and the basic level of cell oxidative metabolism is higher (Yang et al., 2023). The Kupffer cells here are derived from embryos and long-term settled in liver tissue. They highly express various pathogen recognition receptors and act as an immune defense line in the blood vessels to specifically remove pathogenic bacteria and macromolecular antigens from the intestine that enter the liver with the blood (Gomez Perdiguero et al., 2015). Neutrophils will be in a hyperoxia environment after entering the hepatic sinus around the portal vein. This environment can greatly enhance the oxidative burst function of cells. Once the tissue damage-related signals are received, the activation reaction can be quickly initiated.

In contrast, Zone 3 itself lacks oxygen supply and is an active area of lipid metabolism. When thebody has metabolic-related steatohepatitis, alcoholic liver disease and other lesions. Hepatocytes in Zone3 will suffer from strong lipotoxic damage, balloon-like degeneration, and even ischemic necrosis (Leow et al., 2023). Metabolic stress stabilizes HIF-1α protein in hepatocytes and surrounding myeloid cells, prompting cell metabolism to switch to glycolysis mode. The spatial transcriptome data showed that the damaged hepatocytes in Zone 3 specifically activated multiple chemokine signaling pathways, among which the upregulation of CCL2/CCR2 and CXCL1/2/CXCR2 was the most prominent (Chen G. et al., 2026). Affected by the characteristics of hepatic lobule partition, inflammatory monocytes with high expression of Ly6 C and neutrophils carrying CXCR2 in the blood will accumulate in a large number of necrotic lesions around the central vein, forming a local strong pro-inflammatory immune microenvironment (Kim and Lachmann, 2026). High-throughput spatial multi-omics data show that the cell recruitment effect in the peripheral area of the central vein is furtheraggravated by endothelial activation. Long-term hypoxia can promote the expression of ICAM-1 and VCAM-1 in the sinusoidal endothelium of zone 3, forming a dedicated molecular channel, which facilitates the migration of myeloid cells from the blood vessels to the parenchyma of liver tissue (Wa et al., 2025).

With the help of 10x Visium, CosMx spatial molecular imaging and other platforms, high-precision spatial transcriptome studies have been carried out. It has been verified by clinical samples that the interaction axis of neutrophils and macrophages will be strictly distributed according to the hepatic lobule partition (Bravo González-Blas et al., 2024). Spatial mapping reveals that the periportal area (Zone 1) maintains a hyperoxic, nutrient-rich microenvironment characterized by high expression of E-cadherin and macrophage antigen-presenting signatures. Myeloid cell interactions in the Zone 1 mainly play a role in restrictive immune surveillance. On the contrary, the three areas around the center have severe hypoxia, high lipid content, and lower blood flow shear force, forming a unique metabolic microenvironment. Various spatial omics data show that under the regulation of HIF-1α, hepatic sinusoidal endothelial cells in Zone 3 will express a large number of specific adhesion molecules, such as a screening channel, which specifically intercepts highly reactive and glycolytic-dependent neutrophils (Paolini et al., 2025). The partitional metabolic characteristics of the hepatic lobule will reshape the transcriptome of the *in situ* Kupffer cells in the Zone 3 and transform them into lipid/scar-related phenotypes that simultaneously express TREM2 and CD9. Once these cells bind to NETs released by neutrophils, they will locally initiate signal transduction and promote fibrosis and pyroptosis.

### 4.2 Zone-specific macrophage-neutrophil interactions

The internal space of the hepatic sinusoid is narrow, and the immune cells rely on direct contact and short-range paracrine signals to complete local interaction. The main cell pairings in the one region are circulating neutrophils and resident Kupffer cells that stably express CLEC4F^+^ and TIM4, which constitute the core communication system in the region. When the liver suffers from chronic basic lesions superimposed with acute infection or aseptic injury, the Kupffer cells in the portal area will transfer tissue fragments to neutrophils by means of adhesion molecules, so as to maintain local immune homeostasis or quickly remove the damage products (De Muynck et al., 2023).

During the development of chronic liver disease, fibrosis and necrosis are prone to occur in Zone 3, where the signal exchange between cells will also undergo pathological changes. Macrophages differentiated from peripheral monocytes accumulate in the periphery of hepatocytes with lipid accumulation and ballooning degeneration, forming a typical coronary structure. At the same time, cells highly expressTREM2 and CD9 and transform into lipid-associated macrophages (Zhou L. et al., 2024). Neutrophils migrating to hypoxiaZone 3 will release a large amount of reactive oxygen species, and modify histones with PAD4 enzyme to form NETs network structure. The results of spatial tracing showed that NETs were distributed in the central hepatic sinus like reticular scaffolds, which could intercept the inflammatory factors such as Interleukin-1 beta (IL-1β) and Tumor Necrosis Factor-alpha (TNF-α) secreted by peripheral lipid-associated macrophages, and significantly increase the concentration of local inflammatory factors.

It is worth noting that this close positive feedback regulation, in addition to paracrine signals, also depends on the direct binding of ligands to receptors. Single-cell spatial localization data confirmed that TREM2^+^ lipid-associated macrophages in Zone 3 synthesize osteopontin (encoding gene Spp1) in large quantities; the protein can directly bind to the integrin receptor on the surface of activated neutrophils, significantly prolong the survival cycle of neutrophils and inhibit apoptosis (Feng et al., 2025). The antimicrobial peptide (LL-37) released by neutrophils can directly bind to the formyl peptide receptor 2 (FPR2) on the surface of lipid-associated macrophages, accelerate the assembly of intracellular NLRP3 inflammasome, and induce a large number of mature IL-1β release. This close-range inflammation positive feedback loop will continue to aggravate local toxic damage, resulting in a large number of hepatocyte apoptosis, and simple steatosis will gradually progress to severe steatohepatitis (Lee et al., 2023).

### 4.3 Disruption of cellular zonation and the fibrotic niche

When chronic liver disease develops to severe fibrosis and cirrhosis, the original complete structureof hepatic lobules is completely destroyed, and the liver immune microenvironment is also reconstructed. After tissue remodeling, hepatic sinus capillarization occurs, and a large number of extracellular matrix (ECM) proteins accumulate. Early lesions are mostly confined to the periphery of the portal triad or the periphery of the central vein, forming perisinusoidal fibrosis and central fibrosis, respectively. Scar-associated macrophages with high expression of CD9 and TREM2 in the neonatal fibrous septum are closely adjacent to activated hepatic stellate cells in space, and the signaling pathways of the two types of cells can form an interactive linkage.

High-precision spatial imaging results showed that neutrophils would continue to accumulate in the dense collagen fiber bundle area. A number of spatial multi-omics studies have confirmed that scar-associated macrophages in fibrotic lesions can secrete a large amount of transforming growth factor β1 (TGF-β1) and CXCL8 (71). After neutrophil infiltration to fibrosis lesions, transdifferentiation reprogramming occurs under the combined action of various cytokines, and Arg1 and vascular endothelial growth factor A (Vegfa) are expressed in large quantities, which differentiate into pro-fibrotic specific cell phenotypes. Such neutrophils adapted to the microenvironment of the lesion can not only secrete matrix metalloproteinases such as Matrix Metalloproteinase 8 (MMP-8) and Matrix Metalloproteinase 9 (MMP-9) to modify the extracellular matrix, but also release arginase-1 to consume local L-arginine and inhibit cytotoxicT cells to exert killing function. The cells are confined within the fibrous septum, thus forming a protective lesion microenvironment that is immunosuppressive and conducive to the continuous progression of fibrosis (Munder, 2009). A large number of collagen fibers wrap up the gathered myeloid cells, blocking the anti-inflammatory signals that can alleviate inflammation and acting on damaged tissues, thus forming a vicious circle: extracellular matrix continues to accumulate, and liver cell damage continues to increase.

The distinctive microenvironmental factors, cellular phenotypes, and localized crosstalk mechanisms distinguishing Zone 1 and Zone 3 during the progression of chronic liver disease are systematically summarized in Table 3.

**TABLE 3:** Comparison of immune microenvironment and cellular characteristics between zone 1 and zone 3 in CLD.

Feature category	Zone 1 (Periportal Immune Niche)	Zone 3 (Pericentral Immune Niche)
Microenvironmental Milieu	Hyperoxic (65mmHg, oxygentension), nutrient-rich, high concentration of gut-derived antigens via portal vein	Hypoxic (30–35 mmHg oxygen tension), nutrient-depleted, rich in lipid metabolites and metabolic waste products
Predominant myeloid lineages	Embryonic-derived resident Kupffer cells (CLEC4F+, TIM4+, VSIG4+); transient, patrolling baseline neutrophils	Recruited monocyte-derived macrophages (MoMFs, Ly6C^high); TREM2+CD9+ lipid-associated macrophages (LAMs); dense infiltrates of PAD4+ CXCR2+neutrophils
Primary metabolic pathways	Active mitochondrial oxidative phosphorylation (OXPHOS), fatty acid beta-oxidation, gluconeogenesis	HIF-1α-driven aerobic glycolysis, active lipogenesis, blocked tricarboxylic acid (TCA) cycle with succinate/citrate accumulation
Chemokine gradient	Driven largely by baseline homeostatic surveillance factors	Marked upregulation of stress-induced chemokine axes (CCL2–CCR2, CXCL1/CXCL2–CXCR2)
Neutrophil phenotype and function	Pre-activated by hyperoxia, exhibit enhanced baseline phagocytosis and superior oxidative burst capacity upon activation	Hypoxia-adapted, prolonged survival time, high rate of PAD4-dependent neutrophil extracellular traps (NETs) formation and ROS-driven cytotoxicity
Macrophage phenotype and function	Immune-tolerogenic or antigen-presenting sentinel state; highly efficient pathogen clearance without robust inflammation	Pro-inflammatory, high NLRP3 inflammasome assembly, copious secretion of mature IL-1β, IL-18, and TNF-α around ballooned hepatocytes
Crosstalk dynamics	Brief, touch-and-go physical interaction between patrolling neutrophils and resident KCs for systemic antigen screening	Sustained, forward-feeding interaction loop; NETs concentrate LAM-derived cytokines; ROS worsens macrophage lipid overload and lysosomal stress
Pathological contribution in CLD	Involved in initial pathogen handling or localized portal tract inflammation during autoimmune/biliary injuries	Core driver of sterile inflammation amplification, severe lipotoxicity, pericentral hepatocyte necrosis, and initiation of pericentral fibrogenesis

### 4.4 Spatial regulation within the subcapsular microenvironment

In addition to the classic zoning rules inside the hepatic lobule, the latest spatial transcriptome map also clarifies the special status of the subcapsular region of the liver: the immune microenvironment of this layer of anatomical boundary is independent of the internal regulatory system of the liver. Relevant spatial experimental data show that the mechanical tension brought by the capsule, coupled with the continuous stimulation of abdominal secretions and bacteria that migrate from the intestine through the abdominal cavity to the liver, will jointly reshape the transcriptional expression pattern of macrophages under the capsule, forming unique cell characteristics that are different from other regions in the liver (Sierro et al., 2017). The specific resident macrophages at the capsule rely on the specific chemokine signaling axis to rapidly recruit and migrate neutrophils to gather in the liver capsule area in the early stage of injury, forming a local immune defense line to prevent the spread of pathogens in the abdominal cavity to the liver parenchyma before the initiation of the internal circulation immune system in the liver.

It is worth emphasizing that the spatial recruitment mechanism is not limited to the internal communication of myeloid cells, and the signal interaction between endothelium and immune cells is also indispensable. Hepatic sinusoidal endothelial cells (HSECs) are not only responsible for vascular scaffold function. A number of high-throughput spatial multi-omics studies have confirmed that endothelial cell subsets in different partitions secrete differentiated microenvironment regulatory factors, and actively construct a local microenvironment suitable for the survival of myeloid cells (McConnell et al., 2023). Long-term injury stimulation will change the function of hepatic sinusoidal endothelial cells: the intracellular homeostasis protection pathway is inhibited, and a large number of special vascular secretory factors are secreted. These signals can guide the surrounding scar-associated macrophages to aggregate along the damaged hepatic sinus channel. The hepatic sinusoidal endothelium is equivalent to the upstream vascular regulatory hub, which pre-activates the entire set of myeloid cell interaction pathways before the immune cells penetrate the vascular wall to infiltrate the tissue.

## 5 The core mechanism and pathological effects of neutrophil-macrophage interaction

### 5.1 Direct cell contact mediated signal transduction

Direct physical contact between neutrophils and macrophages is one of the important forms of theirinteraction. At the site of inflammation, the two types of cells achieve stable anchoring through a variety of adhesion molecules, among which integrin and intercellular adhesion molecule interaction are the most critical (Fuhr et al., 2025). Integrin Mac-1 (CD11b/CD18) on the surface of neutrophils can bind to ICAM-1 on the surface of macrophages or endothelial cells, thereby promoting close contact between cells and enhancing cell retention in the inflammatory microenvironment (Zou et al., 2025).

This physical contact not only has structural significance, but also triggers intracellular signal transduction. Mac-1/ICAM-1 interaction can activate Src family kinase, MAPK and NF-κB signaling pathways in macrophages, thereby up-regulating the expression of pro-inflammatory cytokines and enhancing theinflammatory response (Dong et al., 2025). In addition, the CD44-hyaluronic acid axis and integrin-mediated mechanical signal transduction are also involved in the regulation of cytoskeleton remodeling and cell activation, thereby further strengthening the functional coupling between cells (Sadeghi et al., 2025).

The process of phagocytosis and clearance of neutrophils by macrophages after apoptosis is anotherkey way for direct communication between cells. In the normal physiological environment, macrophagescan recognize ‘pro-phagocytosis signals’ such as phosphatidylserine, phagocytize and degrade apoptoticneutrophils, trigger the anti-inflammatory response, and help the inflammation gradually dissipate (Xiao et al., 2024). However, in chronic liver disease, persistent inflammation, oxidative stress, and impaired phagocytosis of receptors (such as downregulation of MerTK signaling) can lead to decreased efferocytosis efficiency, secondary necrosis of apoptotic cells, and release of a large number of DAMPs, which in turn reverse activate macrophages and amplify the inflammatory response (Du et al., 2023).

### 5.2 Soluble medium-mediated bidirectional network regulation

In addition to direct contact, neutrophils and macrophages also form a complex two-way regulatory network through a variety of soluble mediators, in which cytokines, chemokines and lipid mediators together constitute a signal transduction system.

Neutrophils can release a variety of pro-inflammatory factors, such as TNF-α, IL-1β and Interleukin-17 (IL-17), which induce macrophages to transform into pro-inflammatory phenotype (Wu et al., 2025). At the same time, chemokines such as CXCL8 secreted by cells will continue to recruit more monocytes, building a constantly expanding positive feedback loop of inflammation. In addition, the elastase and other protein products released by neutrophil degranulation can not only directly regulate the functional activity of various cytokines, but also degrade the anti-inflammatory related molecules *in vivo*, and finally change theinflammatory balance of the local microenvironment (Cambier et al., 2023).

In contrast, macrophages, by secreting factors such as CCL2, Macrophage Colony-Stimulating Factor (M-CSF) and Granulocyte-Macrophage Colony-Stimulating Factor(GM-CSF), not only promote monocyte recruitment, but also prolong the survival time of neutrophils and delay their apoptosis (Kumar et al., 2022). In addition, macrophages also secrete inflammatory factors such as Interleukin-6 (IL-6) and Interleukin-23 (IL-23), which can further enhance the activity of neutrophils, thus making the inflammatory response more intense and persistent (Kyriakopoulos et al., 2021).

In recent years, people have gradually begun to pay attention to the role of lipid mediators in the interaction between neutrophils and macrophages. Prostaglandin E2 (PGE2) and leukotrienes, for example, can regulate the activation of neutrophils (Lin et al., 2026), Lipid mediators such as resolvin, which can help inflammation subside, can also promote the apoptosis of neutrophils and enhance the clearance ability of macrophages. It can be seen that the regulation of inflammation in the human body not only depends on protein signals, but also involves a complex lipid signal regulatory network (Jiao et al., 2023).

In addition, extracellular vesicles (EV) -mediated signal transduction is another important way of intercellular information transmission. The extracellular vesicles secreted by neutrophils can encapsulate microRNAs such as miR-223 and miR-146 a, and transport them into macrophages to inhibit the activation of NLRP3 inflammasome and regulate the level of inflammation in the body (Zhu et al., 2022); In contrast, extracellular vesicles released by macrophages carry a variety of inflammatory and metabolic-related signaling molecules, which can regulate the degree of chemotaxis and aggregation of neutrophils and change theiractivation status.

### 5.3 NETs-mediated key interaction axis

NETs are key mediators that mediate the functional interaction between neutrophils and macrophages (Rasmussen and Hawkins, 2022). In the process of chronic liver disease, the body continues to produce a large number of NETs but cannot be cleared in time. This imbalance is the core mechanism for the continuous aggravation of liver inflammation and the continuous progress of fibrosis.

The generation of NETs requires PAD4 to mediate histone citrullination and chromatin depolymerization, accompanied by reactive oxygen species generation and granular protein release (Wang S. et al., 2024). NETs are mainly composed of DNA network scaffolds, histones and a variety of antibacterial proteins. The pattern recognition receptors such as TLR4 and TLR9 on the surface of macrophages can recognize various components of NETs and determine them as damage-related molecular patterns, thereby activating the NF-κB pathway and NLRP3 inflammasome, and promoting the secretion of various pro-inflammatory factors such as IL-1β and Interleukin (IL-18) (Zhan et al., 2023).

In addition, NETs can also serve as a“scaffold”for a variety of inflammatory signals, centrally presenting DAMPs and cytokines, thereby enhancing local inflammatory responses. Histones and proteases in NETs can also directly damage liver cells and endothelial cells, further aggravating tissue damage (Block et al., 2022).

There is a positive regulatory loop between NETs and macrophages, and this mechanism is particularly critical. Macrophages release inflammatory mediators such as IL-1β, which in turn stimulates neutrophils to produce more NETs, and the two interact to form a continuously amplified inflammatory positive feedback loop (Mousset et al., 2023). In addition, NETs can also act on hepatic stellate cells and activate them, induce cells to transform into myofibroblasts, and accelerate the deposition of collagen, thus opening up the intermediate link of the development of inflammatory response to fibrosis (Xia et al., 2025).

### 5.4 Functional coupling mediated by metabolism and redox state

In addition to classical inflammatory signals, metabolic regulation and redox state also play a central role in the interaction between neutrophils and macrophages. Neutrophils produce a large amount of reactive oxygen species through the NADPH oxidase system during activation, which not only directly damages the liver cells, but also changes the local redox environment, thus affecting the metabolic pattern and functional phenotype of macrophages (Almalki and Almujri, 2024).

Under high oxidative stress, macrophages are more likely to adopt glycolytic metabolism and maintain a pro-inflammatory state (Lin et al., 2024). At the same time, lactic acid accumulation and local hypoxic environment can enhance the expression of inflammatory genes and promote the activation of fibrosis-related pathways by activating HIF-1α signaling (Wu et al., 2024). At the same time, ROS can also act as a signaling molecule, regulate inflammasome activation and pyroptosis and other cell death-related pathways, and continue to aggravate local inflammatory responses (Wu et al., 2021).

In addition, various metabolites produced by macrophages will in turn affect the physiological function of neutrophils. Lipid metabolites, lactic acid and various inflammation-related metabolic intermediates can not only enhance the chemotaxis of neutrophils and promote degranulation, but also change the cell survival cycle and regulate their inflammatory effects (Mihlan et al., 2024; Holter et al., 2025). At the same time, the imbalance of cholesterol metabolism and lipid accumulation will change the cell membrane structure and interfere with intracellular signal transduction, which will aggravate the interaction between various types of cells (Zhou R. et al., 2024).

The metabolic level and redox state mediate two-way regulation, allowing neutrophils and macrophages to work closely together, relying on the metabolic-inflammatory positive feedback loop to continuously break the local microenvironment homeostasis. The core mechanism of the interaction between neutrophils and macrophages is summarized in Table 4.

**TABLE 4:** Mechanisms of crosstalk.

Mechanism	Molecules	Direction	Outcome	Impact
Cell contact	Mac-1/ICAM-1	Bidirectional	NF-κB activation	Inflammation
Cytokines	TNF-α, IL-1β	Bidirectional	Activation	Chronic inflammation
Chemokines	CXCL8, CCL2	Bidirectional	Recruitment	Infiltration
NETs	DNA, histones	Neutrophil→macrophage	Inflammasome	Fibrosis
EVs	miR-223	Bidirectional	Gene regulation	Modulation

### 5.5 Intersections of core signaling pathways and epigenetic-metabolic wiring

The neutrophil-macrophage axis-mediated pathological damage does not rely on a single linear signaling pathway, but a complex cross-linked signaling regulatory network. There are cross-regulations between NF-κB, NLRP3, JAK-STAT, and TGF-β/Smad pathways During sterile inflammation initiation, neutrophil-derived DAMPs and ROS engage TLRs on macrophages, recruiting upstream kinases such as Inhibitor of Nuclear Factor Kappa-B Kinase Beta (IKKβ) and Apoptosis Signal-Regulating Kinase 1 (ASK1). Activated IKKβ drives the phosphorylation and degradation of inhibitor of nuclear factor kappa B alpha (IκBα), liberating canonical NF-κB (p50/p65) to translocate into the nucleus and transcribe pro-IL-1β and NLRP3 components (the priming step) (Shan et al., 2026). At the same time, ROS produced by neutrophils activates ASK1 molecules, initiates downstream MAPK signaling pathways, and greatly increases the transcription level of various inflammation-related genes. After the cells enter the secondary activation and maturation stage, the metabolic signal becomes the key regulatory node: the abnormally activated mammalian target of rapamycin complex 1 (mTORC1) senses the excess nutrients and energy in the cells. On the one hand, it phosphorylates the specific domain of NLRP3, and on the other hand, it maintains the stability of NLRP3 protein. The dual role accelerates the assembly process of inflammasomes (Ma et al., 2020). The assembled complex can be used as a molecular scaffold to recruit ASC and caspase-1; caspase-1 cleaves inactive IL-1β precursor and transforms it into mature, highly toxic and secreted activated IL-1β. In addition, extracellular vesicles released by neutrophils, or the direct binding of intercellular CD11b to ICAM-1, can activate the Janus Kinase 2 (JAK2)- macrophages. Signal Transducer and Activator of Transcription 3 (STAT3) signaling pathway in macrophages. STAT3 is like a molecular regulatory rheostat: short-term activation of STAT3 will promote the secretion of anti-inflammatory factor IL-10, but long-term excessive activation will cooperate with the NF-κB pathway, greatly enhance the transcriptional activity of the NOD-like receptor family pyrin domain-containing 3 (Nlrp3) promoter, promote the assembly of a large number of inflammasomes, and ultimately induce pyroptosis (Wu et al., 2022). When fibrosis progresses to the late stage, macrophages continue to secrete TGF-β1, induce phosphorylation of downstream Smad2/3, directly inhibit the classical NF-κB inflammatory pathway, alleviate acute inflammatory response, and promote neutrophils and macrophages to promote extracellular matrix deposition and mediate functional phenotype of tissue remodeling.

It is worth emphasizing that this cross-signaling pathway is closely bound to the cell‘s own metabolic and epigenetic regulatory systems. A large number of studies have confirmed that macrophages will undergo glycolysis metabolic reprogramming, and the latest single-cell energy metabolism test results show that neutrophils will also switch to glycolysis metabolic mode after infiltrating into the hypoxic liver microenvironment. Abnormal hyperglycolysis causes a large accumulation of intracellular lactic acid, and the role of lactic acid is not limited to metabolites; accumulated lactic acid can act on the nucleus of neutrophils and peripheral macrophages to mediate a new epigenetic modification, namely, histone lactate, of which the 18th lysine of H3 lactated H3K18la is the most typical (Zhang et al., 2019). The epigenetic modification induced by lactic acid can loosen the spatial structure of chromatin. On the one hand, it comprehensively upregulates the transcription levels of pro-fibrotic genes such as Tgfb1 and Arg1, and on the other hand, it inhibits the related genes that maintain immune surveillance homeostasis. Moreover, the continuous release of ROS by neutrophils will increase the expression level of histone methyltransferases such as Enhancer of Zeste Homolog 2 (EZH2), and then change the methylation modification characteristics of Spp1 and Mmp9 gene promoters in macrophages. Metabolism, epigenetics and signaling pathways form a complex linkage regulatory network, which makes cells form a lasting pathological memory and changes the plasticity of cell differentiation, resulting in chronic liver injury that is difficult to subside and repair (Zhao et al., 2025).

## 6 Dynamic stage-specific reprogramming

### 6.1 Molecular checkpoints and switches driving reprogramming

The interaction pattern between neutrophils and macrophages will change periodically with the course of the disease:a feedback loop that amplifies acute inflammation is formed in the early stage, and then switched to a signal network that regulates fibrotic tissue remodeling, and finally a tumor microenvironment with immunosuppressive characteristics is constructed. The evolution process is dominated by various specific molecular checkpoints and local microenvironment thresholds, not simply advancing linearly with the course of disease.

The core regulatory node that determines the transition from the inflammatory initiation stage to the fibrosis process lies in the dynamic balance between cell clearance and cell necrosis. When the body tends to repair inflammation, macrophages recognize and remove apoptotic neutrophils with the help of MerTK receptors, and trigger cell function conversion, secreting a large number of lipid anti-inflammatory mediators such as resolvin and protection and IL-10 (Cai et al., 2016). Hepatocytes release lipid injury-related molecules such as oxidized low-density lipoprotein and cholesterol crystals. Once accumulated to a critical concentration, the MerTK receptor on the surface of macrophages will be cleaved and degraded by proteases, and the protein expression level will be significantly reduced. After the failure of the burial clearance mechanism, the neutrophils that are not removed in time will undergo secondary necrosis, releasing a large amount of citrulline-modified histones and high concentrations of IL-1β to the surrounding microenvironment, and the local IL-1β concentration exceeds the critical regulation level of 50 pg/mL (Cai et al., 2020). Persistent stimulation of long-term inflammatory factors will completely change the transcriptional characteristics of macrophages, so that they can stably maintain the phenotype of scar-associated macrophages, and activate peripheral hepatic stellate cells by Smad3 signaling pathway.

The regulatory switch formed by lactic acid and hypoxia accumulation is the second key node, which dominates the progression of the lesion to the tumor-promoting stage. The continuous accumulation of liver fibrosis matrix will gradually lead to hepatic sinusoidal capillarization, tissue oxygen deficiency, and local oxygen partial pressure drop below the critical value of 15 mmHg. At the same time, the concentration of lactic acid in the microenvironment exceeded 1 mM, and the dual stimulation formed a decisive molecular conversion signal, which strongly activated the target genes of lactic acid modification dependent on the HIF-1α pathway. Neutrophils then differentiate into PMN-MDSCs, and macrophages transdifferentiate into TAMs, which together construct a tumor-promoting and immunosuppressive microenvironment (Gao J. et al., 2025). This metabolic threshold will fundamentally block the antigen presentation process and the proliferation of effector T cells, so that the core axis of cell interaction is completely shifted from matrix remodeling to tumor immune escape.

### 6.2 Inflammation initiation stage: acute reaction and inflammation amplification

In order to clarify the primary and secondary causal logic of myeloid cell interaction in the complete pathogenesis of chronic liver disease, it is necessary to strictly distinguish between early initiating factors and subsequent media molecules that play a role in feedback regulation. According to the order of lesion development, the liver‘s inherent Kupffer cells are the first immune sentinels to respond, and are also the starting source of this cell interaction system. Within a few minutes after acute aseptic injury to the liver, Kupffer cells can sense the lipotoxic stimulation of liver tissue and the DAMPs released by hepatocytes, rapidly activate the transcription of related genes, and preferentially secrete chemokines such as CXCL1, CXCL2, and CXCL8 (113). This kind of early molecular signal is the upstream inducement to initiate the lesion. It recruits a large number of neutrophils in the blood and is also the first immune cell to accumulate after injury. The peak of infiltration occurs from 0 to 24 h after injury. Neutrophils are highly activated after infiltrating the lesion, and rapid degranulation releases ROS. At the same time, PAD4 enzyme catalyzes citrullinated histones to generate NETs (Maali et al., 2024). Various products produced by neutrophils are important secondary stimulation signals, which can bind to TLR4 and TLR9 receptors on the surface of infiltrating mononuclear macrophages, greatly activate the classical pathway of NF-κB and NLRP3 inflammasome in cells, and promote the release of mature IL-1β, TNF-α and other pro-inflammatory factors. The whole set of orderly reaction chain realizes the complete connection from early damage identification to continuous amplification of inflammation.

The most primitive physiological function of this reaction stage is to quickly establish a sterile injury defense system. Activated macrophages secrete TNF-α, IL-1β and other factors to prolong the survival cycle of neutrophils, thus forming a typical sequential positive feedback cycle. At the same time, inflammatory mediators such as IL-1β will in turn promote the formation of NETs and continuously amplify local inflammatory signals.

If the inflammatory phase continues for a long time, the inflammatory regression control node cannot be started in time, and the body‘s steady-state clearance function will gradually be exhausted. Only when apoptotic neutrophils are successfully cleared and subsequently activate anti-inflammatory-related signals, local inflammation will gradually transition to the regression stage (Wang X. et al., 2023). Once the clearance mechanism of apoptotic cells is impaired, or the inflammatory stimulation persists, the positive feedback loop of inflammation will continue to be activated, prompting the neutrophil-macrophage interaction system to gradually evolve to the stage of chronic fibrosis (Poon et al., 2024).

### 6.3 Stage of fibrosis progression: immune-matrix remodeling coupling

As inflammation has not improved and persists, the liver will slowly enter the stage of fibrosis. In this process, the interaction between neutrophils and macrophages is no longer simply amplifying inflammation, but becomes a state of deep integration of immune response and tissue remodeling. The key point is that hepatic stellate cells are continuously activated to promote the development of fibrosis (Wang F. et al., 2024).

NETs produced by neutrophils, as well as proteases such as neutrophil elastase and matrix metallo proteinases (MMPs), can directly affect hepatic stellate cells. They promote the transformation of hepatic stellate cells into the phenotype of myofibroblasts, and allow cells to synthesize more collagen, further promoting liver fibrosis (Jia et al., 2024). At the same time, NETs can also activate the NLRP3 inflammasome pathway in macrophages, promote macrophages to secrete more pro-fibrotic signaling molecules, and continue to promote the progression of liver fibrosis (Artlett, 2022).

A large number of SAMs accumulate in liver fibrosis lesions. These cells secrete a variety of pro-fibrotic factors such as TGF-β and PDGF, which continuously stimulate hepatic stellate cells to maintain activation and accelerate the process of fibrosis (Osna et al., 2022). In addition, SAMs can also directly regulate the function of hepatic stellate cells through Notch and Wnt pathways, and build a stable signal regulatory network between immune cells and stromal cells (He et al., 2024).

It is worth noting that metabolic factors also play an important regulatory role at this stage. For example, lactic acid accumulation and lipid metabolism disorders can enhance the fibrotic phenotype of HSCs and promote macrophages to maintain a pro-fibrotic state, thus forming a positive feedback loop of “metabolism-immunity-matrix” (Li X. et al., 2025).

Previous studies can only qualitatively describe the relationship between immune cells and extracellular matrix. Nowadays, in order to achieve in-depth analysis at the quantitative level, more and more frontier studies combine multi-omics joint analysis and computational simulation, such as single cell differentiation trajectory deduction and spatial localization ligand receptor interaction network analysis. Based on this mature bioinformatics analysis scheme, researchers have been able to quantify the dynamics of macrophage differentiation in fibrotic lesions and the critical regulatory range of interactions between different myeloid cells. Researchers used quantitative cell trajectory modeling to analyze the precise intercellular signal communication network during the formation of metabolic-related fibrosis. Through computational network simulation, it is verified that the adhesion pathway formed by Selplg and Sell (P-selectin glycoprotein ligand one binding L-selectin) is a key quantitative driving factor, which can firmly adhere highly activated neutrophils to the surface of phenotypically remodeled macrophages. The close contact of cells will activate the downstream mechanical transduction pathway, significantly upregulate the YAP signal in myeloid cells, promote macrophages to favor the pro-fibrotic SAM-like functional phenotype, and multiply the collagen deposition ability of adjacent hepatic stellate cells (Nian et al., 2026). For other causes of liver disease with low incidence, quantitative calculation simulation with the same accuracy cannot be carried out at this stage, which also establishes an important research goal for subsequent *in vivo* experiments: to accurately determine the stoichiometric critical range of myeloid cell interaction.

### 6.4 Inflammation regression and tissue repair stage: functional turn and homeostasis reconstruction

Sometimes, if the pathogenic factors leading to liver disease can be effectively controlled, the liver will gradually enter the stage of inflammation regression and tissue repair. In this process, the function of neutrophils and macrophages will slowly change - no longer to promote inflammation, but to anti-inflammatory and help tissue repair. The core driving reason is that the process of eliminating apoptotic cells (efferocytosis) has become more efficient, and anti-inflammatory-related signaling pathways have also been activated.

Macrophages will phagocytose apoptotic neutrophils, which will transform themselves into anti-inflammatory cell phenotypes, and will also make anti-inflammatory factors such as IL-10 and TGF-β, as well as resolution-related signaling pathways become more active. In this way, it can not only inhibit the inflammatory response, but also help the immune system to restore a stable state. At the same time, this phagocytosis process can also increase the expression of matrix metalloproteinases, which can promote the degradation of extracellular matrix and help reverse the already formed liver fibrosis (Wang Z. et al., 2023).

At this stage of tissue repair, neutrophils will also undergo functional changes. It can secrete matrix metalloproteinases MMPs, involved in liver tissue repair and reconstruction; it can also release extracellular vesicles carrying a large amount of miR-223, which is used to inhibit the activation of inflammasomes in macrophages and help inflammation slowly subside. In addition, some neutrophils synthesize anti-inflammatory lipid substances, which are involved in the process of stopping inflammation and ending the inflammatory response (Zhang M. et al., 2023).

However, the process of liver repair is particularly ‘picking the environment'-if there are still problems such as lipid toxicity and hypoxia in the body, or if the inflammatory stimulation does not completely disappear, the repair process often cannot be fully initiated. Therefore, inflammation and fibrosis will always exist at the same time, and the liver cannot return to normal smoothly.

### 6.5 Etiology-specific variations in myeloid axis regulation

The basic etiology of chronic liver disease will greatly affect the interaction between neutrophils and macrophages. There are significant differences between the initiating molecules corresponding to different causes and the follow-up intervention ideas. In the case of viral hepatitis such as chronic hepatitis B and chronic hepatitis C, the whole cell interaction system is mainly regulated by the interferon pathway and the systemic spread of pathogen-associated molecular patterns (PAMPs). Macrophages express a large number of interferon-stimulated genes to exert antiviral effects, and continue to recruit neutrophils to clear virus-infected hepatocytes, but this process often mistakenly injures surrounding normal liver tissues (Krenkel and Tacke, 2017). In contrast, the pathogenesis of MASH is rooted in lipotoxic stimulation, massive accumulation of free fatty acids, and lysosomal functional stress in the Zone 3. This special microenvironment specifically induces macrophages to differentiate into TREM2-positive LAMs, which then interact with highly glycolytic and continuously activated neutrophils (Hou et al., 2021). Alcoholic liver disease will bring double superimposed damage: First, the intestinal barrier is damaged, and lipopolysaccharide enters the liver through the intestinal-liver axis, inducing severe endotoxemia; second, ethanol directly stimulates liver cells and induces oxidative stress. Double stimulation can cause a large number of neutrophils to infiltrate rapidly and generate a large number of NETs, and the clearance ability of liver *in situ* Kupffer cells is difficult to load.

The characteristics of lesions corresponding to various causes directly determine the clinical intervention plan. For MASH, the primary goal of treatment is to correct systemic metabolic disorders, such as the use of Farnesoid X Receptor (FXR), Peroxisome Proliferator-Activated Receptor (PPAR) agonists, and the elimination of lipotoxic stimuli that activate myeloid cell pathways.; in order to achieve the ideal effect of alcoholic liver disease, on the one hand, it is necessary to repair the damaged intestinal mucosal barrier, on the other hand, it is necessary to remove or inhibit pathological NETs in time to prevent a large number of neutrophils from releasing inflammatory substances (Steinberg et al., 2025). For viral liver disease, while carrying out antiviral therapy, targeted immunomodulatory drugs such as JAK inhibitors need to be combined to moderately alleviate the damage caused by the continuous attack of myeloid cells on surrounding normal liver cells, while retaining the body‘s basic immune function against the virus.

### 6.6 Tumor stage: immunosuppression and microenvironment remodeling

Under the continuous action of long-term chronic inflammation and fibrosis, the liver can eventually progress to hepatocellular carcinoma. In the tumor microenvironment, the interaction pattern between neutrophils and macrophages has undergone a fundamental change, switching from an early pro-inflammatory state to an immunosuppressive and tumor-promoting functional phenotype.

Neutrophils can differentiate into PMN-MDSCs, which inhibit the function of CD8^+^ T cells by expressing Arg-1, producing ROS and reactive nitrogen substances, and depleting local arginine (Zeng et al., 2023). In addition, it can also secrete chemokines and proteases to promote tumor cell invasion and metastasis. At the same time, macrophages further differentiate into tumor-associated macrophages (TAMs), which inhibit anti-tumor immune responses by secreting IL-10, TGF-β and expressing PD-L1 (Hao et al., 2022). In addition, TAMs can further inhibit the function of effector T cells by regulating the metabolic microenvironment (such as enhancing lactic acid accumulation) (Li et al., 2023).

The two types of cells still maintain close interaction. For example, cytokines secreted byTAMs can promote the transformation of neutrophils into immunosuppressive phenotype, while ROS and inflammatory mediators produced by PMN-MDSCs can further stabilize the immunosuppressive state of TAMs, thus forming an ‘immunosuppressive positive feedback loop’ (Mabrouk et al., 2023).

In addition to the two-way pro-inflammatory cycle between neutrophils and macrophages, they also interact with adaptive immune cells and various innate lymphocytes in the tumor microenvironment to synergistically construct a larger cell regulatory network (Anitua et al., 2026). The cell-binding structure and soluble factors produced by this cell-cell interaction system will directly promote the functional impairment and depletion of CD8 T cells in advanced fibrosis lesions and liver cancer tissues. PMN-MDSCs can competitively consume local nutrition and directly block T cell receptor signal transduction. TAMs secrete TGF-β and PD-L1, which induce effector T cells to form an irreversible depleted transcriptional phenotype. At the same time, the interaction of myeloid cells also suppresses the anti-tumor surveillance function of NK cells: ROS produced by neutrophils combined with inhibitory factors released by macrophages jointly reduce the level of activated receptors such as Natural Killer Group 2 Member D (NKG2D) on the surface of Natural Killer cells(NK), greatly reducing their ability to kill tumor cells (Bronte and Zanovello, 2005). Conversely, phenotypically altered B cell subsets and plasma cells in the surrounding tissues of the tumor secrete regulatory immunoglobulins, which further stimulate macrophages to differentiate into highly invasive TAMs. The regulatory network formed by the interweaving of multi-layer immune cells is sufficient to show that the interaction system between neutrophils and macrophages is the core center for regulating immune escape in the whole liver tumor tissue.

In addition, neutrophils and macrophages can also secrete pro-angiogenic factors such as vascular endothelial growth factor (VEGF), matrix remodeling enzymes, and promote tumor angiogenesis and tissue invasion (Lorenc et al., 2024). The core features of this stage are impaired immune surveillance and the formation of tumor-promoting microenvironment, which plays a decisive role in tumorigenesis and progression. Stage-specific roles of neutrophil–macrophage interaction are summarized in Table 5.

**TABLE 5:** Stage-specific roles.

Stage	Neutrophil	Macrophage	Crosstalk	Outcome
Initiation	ROS, NETs	Cytokines	Positive loop	Inflammation
Fibrosis	Proteases	SAMs	HSC activation	ECM
Resolution	MMPs	Efferocytosis	Anti-inflammatory	Repair
Tumor	PMN-MDSCs	TAMs	Suppression	Cancer

## 7 Therapeutic strategies

### 7.1 Chemotaxis intervention: blocking immune cell recruitment

The recruitment process of neutrophils and monocyte-derived macrophages depends on the specific chemokine signaling axis. Therefore, targeting chemokines and their receptors has become one of the important therapeutic ideas. Among them, CCR2-CCL2 and CCR5 related pathways play a key regulatory role in monocyte recruitment, inflammation maintenance and fibrosis progression (Guo et al., 2023).

Early clinical studies on MASH found that CCR2/CCR5 dual target antagonist Cenicriviroc (CVC) showed good anti-fibrotic effect in the phase II clinical trial of CENTAUR. However, in the subsequent phase III AURORA clinical trial, the drug did not reach the preset main histological evaluation index, and the clinical trial was stopped in advance (Russell-Hallinan et al., 2025). Through in-depth retrospective analysis, it can be found that the root cause of the failure of this clinical trial is that the liver chemokine regulatory network has strong functional redundancy and compensatory pathway reconstruction will occur. Once the CCR2/CCR5 pathway is completely inhibited, the liver microenvironment in a highly inflammatory state for a long time will compensatory activate other cell migration pathways. Monocytes and neutrophils in the blood can rely on CXCR2-CXCL1/8, CCR1 and other pathways to avoid drug blocking, making the treatment that targets only a single pathway completely ineffective (Rafaqat et al., 2025).

In order to overcome the limitations of single-target drugs, the current transformation research focuses on the development of candidate drugs such as dual-target preparations that can act on myeloid cells, and many drugs have entered the clinical trial stage. The pan-caspase inhibitor Emricasan and the CCR2/CCR5 dual antagonist BMS-817378, which are structurally optimized and more suitable for combination therapy, are currently undergoing comprehensive clinical evaluation. The two drugs can simultaneously block cell chemotaxis, inhibit pyroptosis, and exert dual liver protection (Harrison et al., 2020). Clinical trials of CXCR1/CXCR2 dual-target antagonists such as Ladarixin and Reparixin have been carried out for aseptic inflammation-related diseases. These drugs can avoid the compensatory accumulation of neutrophils, while retaining the body‘s basic immune surveillance ability, and will not completely inhibit the innate immune defense function.

In addition, the strategy of targeting CXCR2 can effectively inhibit neutrophil recruitment and has been shown to reduce inflammation and fibrosis in animal models. However, the key role of neutrophils in anti-infection limits the clinical application of this strategy, and its potential risk lies in increasing infection susceptibility. Therefore, it is currently more inclined to usechemotactic axis inhibition as part of a combination therapy, such as combined with anti-inflammatory or anti-fibrotic drugs, to achieve finer immune regulation.

### 7.2 Targeted NETs formation and clearance: blocking the core loop of inflammation amplification

NETs significantly amplify the inflammatory interaction between neutrophils and macrophages. Focusing on this core mechanism, how to inhibit the generation of NETs and accelerate its removal is the key research direction in recent years (Mutua and Gershwin, 2021). PAD4 is the core enzyme regulating the formation of NETs. A large number of animal experiments have shown that the use of PAD4 inhibitors can significantly reduce the release of NETs, thereby alleviating liver inflammation and delaying the progression of fibrosis (Shen et al., 2026).

In addition, the degradation of NETs structure by exogenous DNase I can directly reduce its accumulation in tissues, thereby reducing its pro-inflammatory and pro-fibrotic effects (Demkow, 2023; Zhang Y. et al., 2024). Recent studies have also found that enhancing the clearance ability of macrophages to NETs such as promoting nucleic acid degradation or phagocytosis) also has potential therapeutic value (Poli and Zanoni, 2023).

It is important to focus on that the regulation of NETs must take into account the body‘s anti-infection ability and inflammation mitigation effect. If the generation of NETs is completely blocked, it will weaken the body‘s immune defense against pathogens. Therefore, the subsequent research and development ideas will not completely inhibit NETs, but prefer to selectively block pathological NETs (such as targeting the PAD4 pathway), or improve the efficiency of the body‘s removal of NETs (Wang et al., 2021).

Although PAD4 inhibitors such as GSK484 have shown good therapeutic potential in preliminary experiments in mice, there are still huge obstacles to promote clinical application. The main problem is that the drug target specificity is poor and it is easy to cause systemic side effects. The mechanism is that PAD4 not only plays a role in immune cells, but also participates in the basic gene transcription and epigenetic regulation of hematopoietic stem cells, mucosal epithelial cells and other normal cells in addition to the regulation of histone citrullinated modification during NETs formation (Rafaqat et al., 2025). This means that long-term systemic administration of non-specific PAD4 inhibitors will bring a series of security risks:inhibiting bone marrow hematopoietic function, damaging stem cell differentiation potential, and causingextensive mucosal erosion. At the same time, citrullinated histone is an important substance for the human body to resist pathogens. If PAD4 activity is completely inhibited, patients are prone to bacteremia and even fatal opportunistic pathogen infection. For the high-risk group of cirrhosis with impaired intestinal-hepatic barrier, the risk of infection will be further increased (Saha et al., 2019). In order to apply PAD4 inhibitors from laboratory results to clinical practice, it is necessary to develop biomimetic nanocarriers that can target neutrophils, or prodrugs that can be activated at the site of inflammation, so that the drugs can only inhibit PAD4 in the liver lesion area and avoid side effects caused by systemic effects.

### 7.3 Metabolism and nuclear receptor regulation: remodeling immune microenvironment

Metabolic reprogramming is an important mechanism that drives the functional transformation of macrophages. Therefore, targeting the immune metabolic pathway provides a new therapeutic idea for regulating neutrophil-macrophage interaction.

The researchers used various mouse gene editing models to carry out experiments and clearly elucidated the causal regulatory relationship between related pathways. More and more mouse knockout and cell clearance experiments have shown that FXR agonists can dynamically regulate bile acid metabolism and directly block the upstream NF-κB inflammatory pathway in macrophages *in vivo* (Wang et al., 2008). In various rat and mouse models of liver injury, the targeted activation of this pathway by drugs can significantly reduce the synthesis and release of various pro-inflammatory factors in myeloid cells and improve the oxidative stress injury of hepatocytes in the lesion area (Wang et al., 2025). At the same time, PPAR agonists can regulate lipid metabolism pathways in the immune microenvironment. With the help of macrophage-specific knockout animal models, its mechanism of action can be elucidated: Activation of PPAR-γ can reshape the metabolic patterns of human and mouse macrophages, promote cell transdifferentiation into macrophage subsets that use fatty acid oxidation as the main energy supply, maintain tissue homeostasis, and inhibit inflammation (Liu et al., 2021).Clinical human association studies have shown that the severity of tissue lipid accumulation is highly synchronized with the degree of myeloid cell infiltration, but it is necessary to distinguish the primary and secondary effects. Available evidence shows that PPAR agonists only directly reshape the internal metabolic mechanism of macrophages; the relief of the overall aseptic inflammation of the liver is only a subsequent secondary effect, and the drug does not directly act on neutrophils and regulate their function.

In recent years, targeted regulation of key signaling pathways such as HIF-1α, Mammalian Target of Rapamycin (mTOR), and Adenosine Monophosphate-Activated Protein Kinase (AMPK) has also been found to have favorable anti-inflammatory potential. For example, inhibiting the glycolysis process of cells can weaken the pro-inflammatory effect of macrophages; improving the oxidative metabolism function of mitochondria can help the body to restore the immune balance and stabilize the inflammatory state (Huang et al., 2021).

However, it is worth noting that this metabolic intervention is a systemic effect and has different effects on different types of cells. Therefore, subsequent research also needs to develop metabolic regulation methods with stronger cell specificity, so as to accurately regulate the immune response of the body and avoid deviations.

### 7.4 Promoting inflammation resolution: targeting efferocytosis and immune reprogramming

In addition to simply inhibiting inflammation, promoting inflammation regression has become a new treatment concept. Among them, enhancing the efferocytosis of macrophages on apoptotic neutrophils is a key link in restoring the body‘s immune homeostasis (Cho et al., 2023).

Intervention of MerTK and its downstream phagocytosis-related signaling pathways can enhance the clearance efficiency of apoptotic cells by macrophages, promote the transformation of macrophages into anti-inflammatory cells, and secrete a variety of anti-inflammatory cytokines such as IL-10 (Shi et al., 2025). In addition, resolvin, a lipid mediator that promotes the resolution of inflammation, can enhance the clearance ability of macrophages and accelerate the inflammatory process to calm down (Mohammad-Rafiei et al., 2024).

In addition, the treatment schemes such as transforming myeloid cell phenotype and reinfusion of immune cells have developed rapidly, no longer only at the theoretical level, and gradually transformed into clinical application. IIn the past, most of the related studies used cardiovascular disease models for reference. Nowadays, autologous macrophage therapy has entered human clinical trials, showing good safety and therapeutic benefits for severe liver injury. Notably, pioneering clinical scaling demonstrated by Carbó et al. (2021) established the feasibility and safety of delivering histologically pre-conditioned autologous macrophages to patients with advanced liver cirrhosis. Building dynamically upon this cellular foundation, a milestone Phase II clinical trial published by Brennan et al. (2025) demonstrated that gene-edited or phenotype-optimized macrophage preparations can target and aggregate at the site of liver injury, significantly reduce local severe inflammation, and help patients with acute-on-chronic liver failure repair damaged liver tissue. This clinical breakthrough fully demonstrates that, instead of blindly inhibiting the overall infiltration of myeloid cells, it is better to adopt a scheme of *in vitro* transformation of macrophages and then reinfusion - such cells can efficiently decompose fibrotic tissue and simultaneously regulate tissue repair, which is a feasible scheme with great landing value for the treatment of advanced chronic liver disease.

The advantage of this type of strategy is not simply to inhibit the immune response, but to achieve disease control by restoring the “self-regulation ability”of the immune system.

### 7.5 Immune checkpoint and combination therapy strategy: Reshaping tumor immune microenvironment

In the stage of liver cancer, immune checkpoint inhibitors are one of the mainstream clinical therapeutic drugs. However, the inhibitory tumor microenvironment constructed by neutrophils and macrophages will greatly weaken the anti-tumor efficacy of these drugs.

Neutrophil-derived PMN-MDSCs and tumor-associated macrophages can inhibit T cell function through multiple mechanisms, including depletion of arginine, release of ROS, and expression of immune checkpoint molecules (Cai et al., 2025). Therefore, only using immune checkpoint inhibitors alone is often difficult to fully activate the body‘s anti-tumor immune response.

In recent years, multi-drug combination and multi-target synergistic therapy have become a hot research direction. Immune checkpoint inhibitors combined with anti-angiogenic drugs can reshape tumor blood vessels and promote immune cell infiltration, thereby improving the overall anti-tumor effect. At the same time, synchronous intervention of myeloid cells (such as inhibiting Myeloid-Derived Suppressor Cells (MDSCs)recruitment or reprogramming TAMs) can also effectively break through the immunosuppressive barriers of tumor microenvironment (He et al., 2025).

The future treatment strategy is more likely to develop towards“multi-pathway synergistic regulation”, that is, targeting inflammation, metabolism and immunosuppressive networks at the same time, so as to achieve systematic remodeling of complex tumor microenvironment. Therapeutic strategies targeting this axis are summarized in Table 6.

**TABLE 6:** Therapeutic strategies.

Strategy	Target	Mechanism	Advantages	Limitations
Chemokine inhibition	CCR2/CCR5	Block recruitment	Reduce inflammation	Redundancy
NETs inhibition	PAD4	Reduce NETs	Anti-fibrotic	Infection risk
Metabolic modulation	FXR	Reprogramming	Broad effects	Specificity
Efferocytosis	MerTK	Resolution	Homeostasis	Limited data
Immunotherapy	PD-1	Restore immunity	Effective in HCC	Complexity

### 7.6 Critical caveats, contradictory paradigms, and technical limitations

At present, the basic research related to liver myeloid cells is difficult to be applied in batches. The root cause is that the physiological mechanisms of different species are very different, and the experimental conclusions are highly dependent on specific animal models. Most of the fine inflammatory regulatory pathways reviewed in this review are derived from animal experiments such as mice; however, there is a huge difference in evolution between human and mouse myeloid cell systems (Feng et al., 2023). For example, human tissue-resident KCs and MoMFs each have independent transcriptional regulatory networks and characteristic surface markers, which do not fully match the corresponding cell lineage characteristics in mice; at the same time, there are great differences in the peripheral basic reserves, arginase one storage and distribution, and dynamic regulation of human neutrophils compared with mouse neutrophils. In addition, various experimental models commonly used in the study of chronic liver disease (CLD), including carbon tetrachloride (CCl4) poisoning model, methionine-choline deficiency (MCD) diet/high-fat diet model, Mdr2 knockout/knockout model, and bile duct ligation (BDL) model, are all artificially constructed pathological models, which have natural deviations from the natural state of human disease (Yang Z. et al., 2025). All kinds of animal models can only reproduce the single and local pathological features of human chronic liver disease, such as simple chemical toxicity injury, artificial lipid accumulation or acute biliary mechanical obstruction. The above-mentioned single stimulation modeling system cannot simulate the complex regulatory network of human clinical diseases with multi-level, hidden onset and continuous progress over decades. Therefore, it is necessary to maintain a high degree of prudence when applying the conclusions of rodent experiments to the diagnosis and treatment of clinical patients.

Although the interwoven regulatory axis between neutrophils and macrophages is generally considered to be a pathogenic positive feedback driving mechanism for chronic liver disease and fibrosis, there are still some key doubts and contradictory experimental data after objective comprehensive analysis. First, some experimental results are contrary to the theoretical model of one-way synergistic pro-inflammatory (Kim et al., 2021). For example, in the early stage of acute lipotoxicity or toxic injury, pre-invasive neutrophils can secrete specific lipid mediators such as resolvin E1, or release extracellular vesicles rich in microRNAs (represented by miR-223), thereby inhibiting macrophage NLRP3 inflammasome assembly, rather than activating this pathway. This indicates that under certain steady-state conditions, the neutrophil-macrophage regulatory axis can play a role in self-limiting inflammation and even promoting inflammation regression (Calvente et al., 2019). However, there are still some differences in the conclusions of different animal models to determine whether the cell interaction is turned into a chronic pathogenic state or a precise critical condition for the timely regression of inflammation.

In addition, this paper is consistent with most of the existing research viewpoints, and all of themquestion the simplified M1/M2 macrophage polarization model, but there is still an important theoretical limitation: other classification systems mentioned in this paper, namely, LAMs, SAMs and (TAMs, the existing literature is not enough to fully confirm that they are functional, stable and independent cell subsets, which is particularly prominent in human clinical tissue samples (Feng et al., 2023). High-dimensional multi-omics reveals substantial phenotypic blending and intermediate transition states among these subsets; a clear-cut, rigid lineage segregation between a “purely fibrotic” SAM and a “purely metabolic” LAM does not exist *in vivo*. Crucially, as emphasized by Cartwright et al. (2025) in temporal studies of myeloid dynamics, this type of new macrophages and neutrophils activation phenotype is not a stable cell phenotype of terminal differentiation, but a continuous variable state formed by cells regulated by multiple microenvironment signals and relying on high plasticity dynamic adaptation. It is easy to oversimplify the myeloid cell system, exaggerate the stability of cell phenotype, form a new classification inherent cognition, and cover up the real lineage plasticity of human chronic liver injury lesions by using fixed and static abbreviations to define such highly instantaneous and microenvironment-dependent continuous cell lineages (Saldarriaga et al., 2020).

Finally, the existing experimental methods have obvious methodological bottlenecks and technical defects, which greatly reduce the reliability of the above biological mechanism interpretation and transformation research conclusions. First, traditional single-cell RNA sequencing (scRNA-seq) can produce severe enzymatic dissociation deviations, resulting in a significantly lower proportion of intrinsic cells in fragile tissues, and even the loss of human cell signals; mature embryo-derived Kupffer cells, hypersensitive circulation and infiltrating neutrophils are most affected by this problem.In addition, traditional scRNA-seq will completely lose the original spatial location information of cells, and it is difficult to directly reflect the real functional status of cells, such as real-time cell burial efficiency, immediate production level of reactive oxygen species (ROS), and dynamic process of NETs release (He et al., 2021). At present, the field relies on transcriptome characteristics to infer the activation state of myeloid cells, but the biological function of neutrophils is mainly regulated by pre-stored granule proteins, rather than new transcription products. The single-cell resolution proteomics technology for neutrophils is still very scarce, which is still a major research blind spot in this field.

Secondly, although high-resolution imaging spatial transcriptome technology (such as MERFISH, Xenium) and multi-channel imaging platforms try to make up for the above defects, a large number of spatial correlation inferences about the close adjacent distribution and special microenvironment structure of neutrophils and macrophages in the existing literature only rely on mathematical calculations, and have not been verified by single-cell resolution imaging experiments in human clinical tissues (Wa et al., 2025). Third, the *in vivo* detection method of NETs is still controversial. Most of the published studies only rely on indirect semi-quantitative overall detection methods (such as circulating MPO-DNA complexes, free DNA extracts) or *in vitro* static imaging to determine that NETs have a clear pathogenic effect. The specificity of *in vivo* detection of such markers is widely questioned, and there is no standardized technical solution that can directly and quantitatively detect local NETs formation (NETosis) in human liver lesions. This is an important detection difficulty, and it is necessary to maintain a high degree of caution when deriving clear causal associations (Hayden et al., 2021). Therefore, we should objectively adjust our cognition and make it clear that the conclusions of the existing models are still mainly derived before the systematic *in situ* space, cell function and proteomics are directly verified in clinical patient tissues.

### 7.7 Patient Stratification and biomarker-driven myeloid interventions

If the phased myeloid cell intervention program proposed in this paper is to be applied clinically, the core relies on non-invasive biomarkers and transcriptome features to accurately stratify patients. There are a variety of heterogeneous microenvironment areas in the liver of the same patient. Without screening and broad-spectrum use of myeloid cell inhibitors, it may cause serious systemic cross-toxic side effects (Ramach et al., 2019). In order to transform the above research strategy from laboratory to clinical, a set of accurate diagnosis process based on circulating myeloid markers and transcriptome detection panel is needed. For example, patients with significantly elevated levels of serum citrullinated histone H3 (citH3) and free MPO-DNA complexes need to be stratified separately in the rapid initiation phase of inflammation, using drugs that remove pathological) or locally effective PAD4 precursor drugs (Ouyang et al., 2025). In contrast, patients with high expression of TREM2/CD9 in peripheral monocytes and characteristic serum markers of macrophage-derived matrix remodeling can choose macrophage adoptive cell therapy or drugs that specifically enhance cell burial to promote matrix degradation and absorption. With the help of such dynamic marker combinations, the cell stage of the dominant lesion in each patient can be determined in real time, which can ensure that the therapeutic drugs targeting myeloid cells act on the target cells at the right time and accurately (Fabre et al., 2023).

## 8 Future perspectives

Neutrophils and macrophages are the core innate immune cells in the immune microenvironment of chronic liver disease. The dynamic interaction between them runs through every stage of the development of the disease, and in different pathological processes, it also shows obvious characteristics of functional reprogramming (Wen et al., 2021). From the rapid amplification reaction at the beginning of inflammation, to the combination of immunity and matrix remodeling in the fibrosis stage, to the formation of immunosuppressive network in the tumor stage, the ‘combination’ of neutrophils and macrophages is not only the direct executor of the inflammatory response, but also the key regulatory link to promote the progress of the disease step by step.

In recent years, with the rapid development of single-cell sequencing and spatial transcriptome technology, the research on the neutrophil-macrophage axis has become more and more in-depth. The study of cognition is no longer only to see the change of cell number, but to upgrade to the specific functional remodeling level of different cell subsets. However, there are still many key scientific problems unsolved. First of all, the specific distribution and division of labor of each subgroup of neutrophils and macrophages in the specific microenvironment of the liver have not been thoroughly studied. In particular, under the unique structural characteristics of the hepatic lobule partition, the correlation mechanism between the spatial distribution of various types of myeloid cells and their functions still needs to be more systematically analyzed.

In addition, the synergistic regulation mechanism between immune metabolic reprogramming and classical inflammatory signaling pathways has not been fully and deeply studied. Like some metabolic intermediates, they can also act as signaling molecules, participate in the regulation of inflammasome activation, and also determine cell growth, differentiation and fate. However, there is currently no complete and unified theory that can systematically explain this mechanism of action.

In addition, analyzing the exact mechanism of neutrophil training immunity and chromatin accessibility remodeling is a key research direction in the field of myeloid cell research in the future. Most of the existing research systems can only capture the static transient state of neutrophil heterogeneity. How to maintain the epigenetic memory at the bone marrow level and how the memory selectively plays a role after cell infiltration into the tissue are still unclear. The follow-up research system should focus on the construction of neutrophil-specific histone modification maps and single-cell ATAC-seq characteristic maps in patients with different stages of chronic liver disease (Ram-Mohan et al., 2021). After exploring the above epigeneticregulatory nodes, researchers will no longer be limited to simply blocking circulating cells. It is expected to open up new therapeutic ideas to eliminate the pathogenic epigenetic memory of neutrophil precursor cells, reverse their training immune status, and block the systemic inflammatory cascade from the upstream bone marrow source.

In addition, the interaction between neutrophils and macrophages is dynamically changing. However, the critical node (i.e., ‘functional transition threshold’) of functional transition of such cells in different processes of disease has not been clearly defined. For example, where is the key regulatory site for inflammation to change from aggravation to slow regression, how does the pro-inflammatory microenvironment turn into an immunosuppressive state, and what are the driving factors behind it, are all current research hotspots and unresolved scientific problems. If these problems can be solved, the study of these 2 cells can move from simply describing their changes to understanding the specific mechanisms and then moving towards the level of regulating them.

From the technical level, the follow-up study needs to integrate more omics related technologies. For example, combining single-cell transcriptome, spatial transcriptome, proteome and metabolome to construct a multi-dimensional regulatory network of neutrophil-macrophage interaction, so as to achieve a more comprehensive mechanism analysis. In addition, the lineage tracing technology combined with *in vivo* imaging can dynamically observe the transformation process of cell fate. At the same time, it is necessary to carry out research on the characteristics of cells in different stages of disease progression, so as to deepen the research depth and make the experimental conclusions more in line with the real pathological state.

At the clinical application level, the treatment idea for the neutrophil-macrophage regulatory axis has gradually shifted from single-target intervention to multi-channel combined intervention. The research directions with broad application prospects in the future are mainly divided into the following categories: selective inhibition of pathogenic NETs generation; accurately intervene in chemotaxis-related pathways to avoid abnormal aggregation of immune cells; remodeling cell function by targeting immune metabolic pathways; clear apoptotic cells by promoting efferocytosis, promote inflammation subsided.Especially when chronic liver disease progresses to the tumor stage, the combination of myeloid cell-targeted drugs and immune checkpoint inhibitors is expected to improve the therapeutic problems brought by the immunosuppressive microenvironment and further improve the overall therapeutic effect.

However, the current core challenge is not simply to inhibit pathological inflammation, but also to retain the body’s anti-infective ability, which is a problem that all treatment strategies must overcome (Chen L. et al., 2026). Therefore, it is urgent to develop precise intervention methods with cell specificity and disease stage specificity to achieve selective regulation and phenotypic reprogramming of immune regulatory networks, rather than indiscriminately inhibiting the overall immune function of the body.

## 9 Conclusion

**FIGURE 1:**
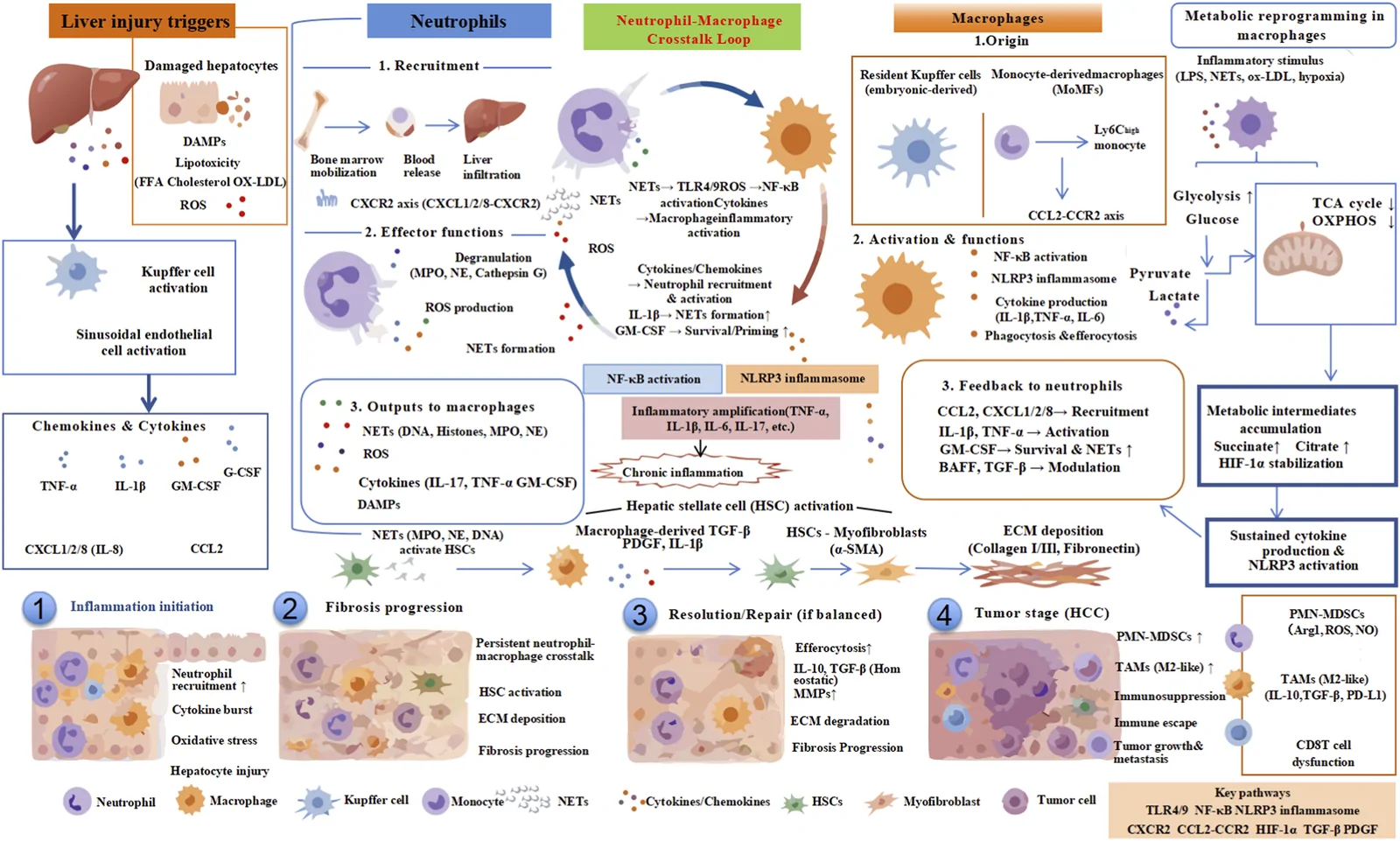
Schematic overview of the spatial immune niche remodeling of the neutrophil-macrophage axis in chronic liver disease. This integrated framework highlights the central role of the neutrophil–macrophage axis in driving inflammation, fibrosis, and tumor progression, and identifies potential therapeutic targets. (The figure was created using BioRender.com.)

In general, the interaction axis between neutrophils and macrophages is like a key“transfer station” connecting inflammatory response, tissue remodeling and tumor immunity. In-depth understanding of their dynamic regulatory mechanisms, as well as the characteristics of functional changes at different stages of the disease, not only allows us to more thoroughly understand the immunopathological process of chronic liver disease, but also provides important theoretical support for the development of new precision treatment methods. The core regulatory network of this neutrophil-macrophage crosstalk in chronic liver disease progression is systematically illustrated in the following schematic diagram (Figure 1). In the future, it is believed that with the increasingly close integration of multi-omics technology and translational medicine research, this field can achieve a substantive breakthrough from understanding the mechanism to clinical practical treatment, bringing new hope to patients with chronic liver disease.

## Glossary

CLD: Chronic Liver Disease
HCC: Hepatocellular Carcinoma
NASH: Nonalcoholic Steatohepatitis
MASH: Metabolic Dysfunction-Associated Steatohepatitis
ALD: Alcoholic Liver Disease
DAMPs: Damage-Associated Molecular Patterns
ROS: Reactive Oxygen Species
NETs: Neutrophil Extracellular Traps
PMN-MDSCs: Polymorphonuclear Myeloid-Derived Suppressor Cells
TAMs: Tumor-Associated Macrophages
KCs: Kupffer Cells
MoMFs: Monocyte-Derived Macrophages
LAMs: Lipid-Associated Macrophages
SAMs: Scar-Associated Macrophages
HSCs: Hepatic Stellate Cells
ECM: Extracellular Matrix
MPO: Myeloperoxidase
NE: Neutrophil Elastase
PAD4: Peptidyl Arginine Deiminase 4
TLR: Toll-Like Receptor
TLR4: Toll-Like Receptor 4
TLR9: Toll-Like Receptor 9
NF-κB: Nuclear Factor Kappa B
NLRP3: NOD-, LRR- and Pyrin Domain-Containing Protein 3
IL: Interleukin
IL-1β: Interleukin-1 Beta
IL-6: Interleukin-6
IL-10: Interleukin-10
IL-17: Interleukin-17
TNF-α: Tumor Necrosis Factor Alpha
TGF-β: Transforming Growth Factor Beta
GM-CSF: Granulocyte-Macrophage Colony-Stimulating Factor
G-CSF: Granulocyte Colony-Stimulating Factor
CXCL1: C-X-C Motif Chemokine Ligand 1
CXCL2: C-X-C Motif Chemokine Ligand 2
CXCL8: C-X-C Motif Chemokine Ligand 8 (Interleukin-8)
CCL2: C-C Motif Chemokine Ligand 2
CCR2: C-C Chemokine Receptor Type 2
CXCR2: C-X-C Chemokine Receptor Type 2
PDGF: Platelet-Derived Growth Factor
PD-1: Programmed Cell Death Protein 1
PD-L1: Programmed Death-Ligand 1
Arg-1: Arginase-1
BAFF: B-Cell Activating Factor
EVs: Extracellular Vesicles
miR: MicroRNA
miR-223: MicroRNA-223
miR-146a: MicroRNA-146a
OXPHOS: Oxidative Phosphorylation
TCA cycle: Tricarboxylic Acid Cycle
HIF-1α: Hypoxia-Inducible Factor 1 Alpha
α-SMA: Alpha-Smooth Muscle Actin
FFA: Free Fatty Acids
ox-LDL: Oxidized Low-Density Lipoprotein
MMPs: Matrix Metalloproteinases
MerTK: MER Tyrosine Kinase
TREM2: Triggering Receptor Expressed on Myeloid Cells 2
CD9: Cluster of Differentiation 9 (Tetraspanin CD9)
SPP1: Secreted Phosphoprotein 1 (Osteopontin)
CLEC4F: C-Type Lectin Domain Family 4 Member F
TIM4: T-Cell Immunoglobulin and Mucin Domain Containing 4
HSECs: Hepatic Sinusoidal Endothelial Cells
LL-37: Cathelicidin LL-37
VCAM-1: Vascular Cell Adhesion Molecule-1
ICAM-1: Intercellular Adhesion Molecule-1
HA: Hyaluronic Acid
CXCR4: C-X-C Chemokine Receptor 4
PEG2: Prostaglandin E2
DRAN: Ductular Reaction-Associated Neutrophils
NADPH: Nicotinamide Adenine Dinucleotide Phosphate
VSIG4: V-set and Immunoglobulin Domain Containing 4
LDNs: Low-Density Neutrophils
SPP1: Secreted Phosphoprotein 1
CitH3: Citrullinated Histone H3
mTOR: Mammalian Target of Rapamycin
IKKβ: Inhibitor of Nuclear Factor Kappa-B Kinase Beta
ASK1: Apoptosis Signal-Regulating Kinase 1
JAK2: Janus Kinase 2
STAT3: Signal Transducer and Activator of Transcription 3
CVC: Cenicriviroc
BDL: Bile Duct Ligation
FXR: Farnesoid X Receptor
AMPK: Adenosine Monophosphate-Activated Protein Kinase
NK: Natural Killer
NKG2D: Natural Killer Group 2 Member D
